# Supplementary material for: Phase-resolved measurement and control of ultrafast dynamics in terahertz electronic oscillators
Source: Nat Commun. 2024 Jul 2;15:5358. doi: 10.1038/s41467-024-48782-4 (PMC11219870; doi:10.1038/s41467-024-48782-4)
Supplement: Supplementary file 1 — Supplementary Information [file 41467_2024_48782_MOESM1_ESM.pdf]

Supplementary information

Phase-resolved measurement and control of ultrafast dynamics in terahertz electronic oscillators

Takashi Arikawa[1,2,6*], Jaeyong Kim[3,7], Toshikazu Mukai[3], Naoki Nishigami[4], Masayuki Fujita[4], Tadao Nagatsuma[4] and Koichiro Tanaka[1,5]

[1] Graduate School of Science, Kyoto University, Kyoto, Japan.
[2] PRESTO, Japan Science and Technology Agency (JST), Saitama, Japan
[3] ROHM Co., Ltd., Kyoto, Japan.
[4] Graduate School of Engineering Science, Osaka University, Toyonaka, Japan
[5] Institute for Integrated Cell-Material Sciences (iCeMS), Kyoto University, Kyoto, Japan
[6] Present address: Graduate School of Engineering, University of Hyogo, Himeji, Japan
[7] Present address: Qualitas semiconductor, co, ltd., Seongnam, Gyeonggi-Do, Republic of Korea
Corresponding author *arikawa@eng.u-hyogo.ac.jp

Internal reflection inside the RTD device

There is an unintentional air gap or thin adhesive layer between the silicon lens and the substrate, which produces echo signals due to the internal reflection between the silicon lens/substrate interface and RTD/air interface. The round-trip time is estimated as 26 ps considering the oblique incidence of the THz beam on the RTD due to the tight focusing by the silicon lens. The first echo is seen at around 35 ps in Fig. 1c. In the difference signal (E_{RTD}), this signal is stronger than the direct reflection signal starting from 9 ps. This is because the first echo undergoes the reflection at the RTD/air interface twice and the signal change due to the RTD reflectivity change is bigger. The second echo is small presumably because the THz beam is no longer confined in this cavity structure.

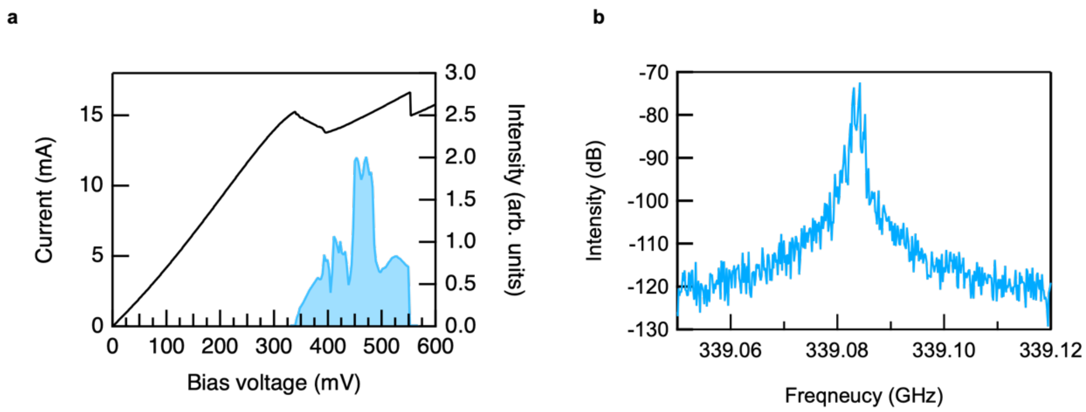

Figure S1 | Basic characterization of RTD THz oscillator. a. Current-voltage and output intensity-voltage characteristics. The RTD was driven by a source meter in voltage source mode. The output intensity is the same one shown in Fig. 2d as blue curve. **b.** Typical spectrum measured by a conventional heterodyne down-conversion method.

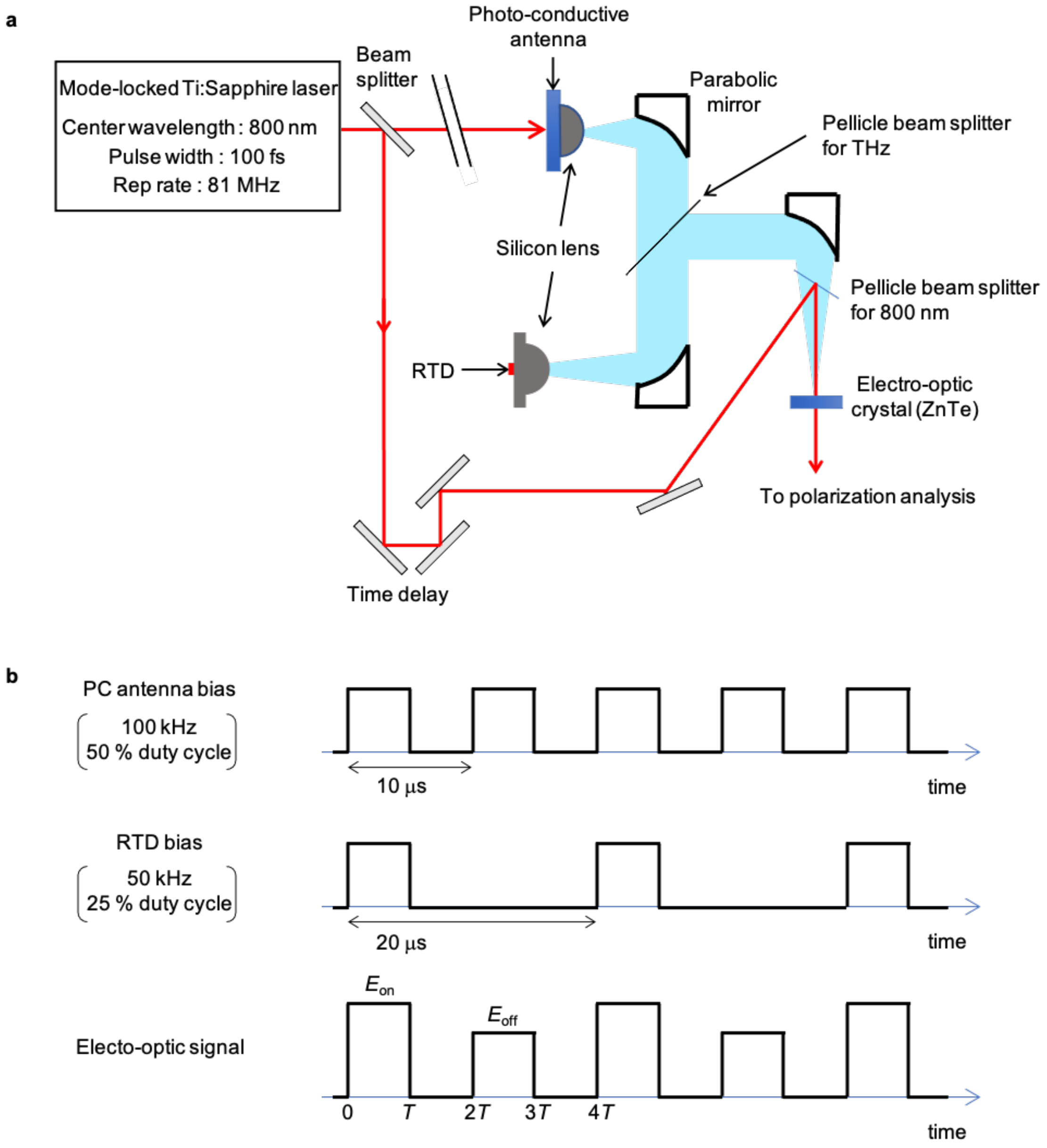

Figure S2 | Experimental setup. a. THz-TDS setup in reflection geometry. **b.** Timing chart for the double modulation.

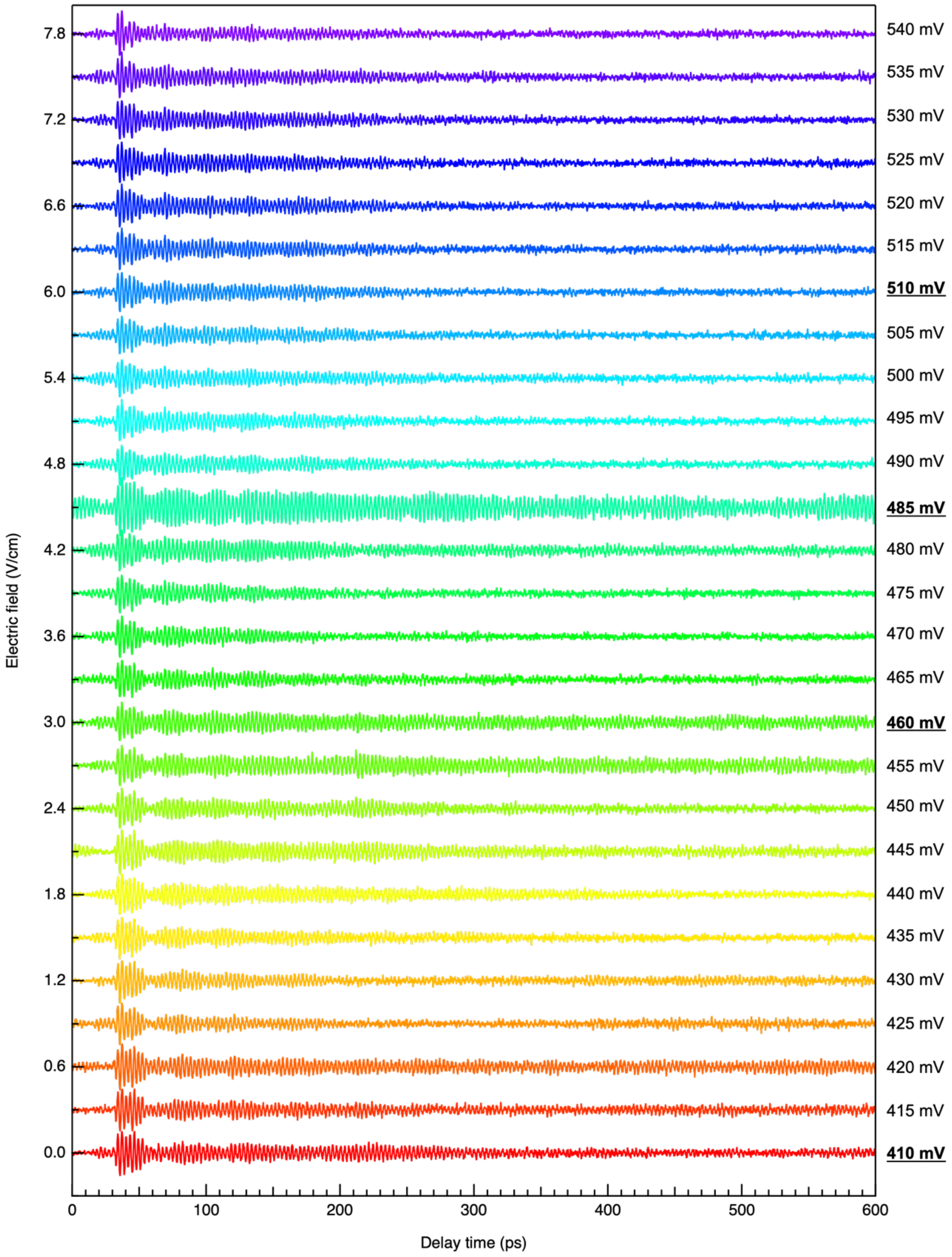

Figure S3| Bias voltage dependence of the difference signal E_{RTD}. The bias voltages of the typical waveforms used in the main text are underlined.
